# A new symmetrodont mammal (Trechnotheria: Zhangheotheriidae) from the Early Cretaceous of China and trechnotherian character evolution

**DOI:** 10.1038/srep26668

**Published:** 2016-05-24

**Authors:** Shundong Bi, Xiaoting Zheng, Jin Meng, Xiaoli Wang, Nicole Robinson, Brian Davis

**Affiliations:** 1 Department of Biology, Indiana University of Pennsylvania, Indiana, PA 15705, USA; 2 Key Laboratory of Vertebrate Evolution and Human Origins of Chinese Academy of Sciences, Institute of Vertebrate Paleontology and Paleoanthropology, Chinese Academy of Sciences, Beijing 100044, China; 3 Shandong Tianyu Museum of Nature, Pingyi, Shandong 273300, China; 4 Institute of Geology and Paleontology, Linyi University, Linyi, Shandong 276000, China; 5 Division of Paleontology, American Museum of Natural History, Central Park West at 79th St., New York, NY 10024, USA; 6 Department of Anatomical Sciences and Neurobiology, University of Louisville, 511 S. Floyd St., Louisville, KY 40202, USA

## Abstract

We report the discovery of *Anebodon luoi*, a new genus and species of zhangheotheriid symmetrodont mammal from the Lujiatun site of the Lower Cretaceous Yixian Formation, China. The fossil is represented by an associated partial skull and dentaries with a nearly complete dentition, and with a dental formula of I4/3 C1/1 P5/4 M3/4. This new taxon lacks the high molar count typical of derived symmetrodonts, differing from the well-represented zhangheotheriids *Zhangheotherium* and *Maotherium* in having a postcanine dental formula that resembles more primitive tinodontid symmetrodonts on the one hand, and sister taxa to therians such as *Peramus* on the other. Upper and lower distal premolars are strongly molariform and are captured undergoing replacement, clarifying positional homology among related taxa. We also describe the rostrum and, for the first time in a symmetrodont, much of the orbital mosaic. Importantly, our new taxon occupies a basal position within the Zhangheotheriidae and permits discussion of trechnotherian character evolution, ultimately shedding additional light on the evolution of therians.

Mesozoic mammals with molariform teeth bearing a simple, triangular arrangement of principal cusps were traditionally assigned to the Order Symmetrodonta^1^. Based originally on taxa from the Upper Jurassic-Lower Cretaceous Morrison and Purbeck formations (e.g., *Spalacotherium*^2^ and *Tinodon*^3^), this classification stood for well over a half century and incorporated subsequently described forms ranging from *Kuehneotherium* (Early Jurassic of Britain^4^) to the pseudotribosphenic *Shuotherium* (Middle Jurassic of China and Britain^5^,^6^,^7^,^8^). Phylogenetic analyses imply that this grouping is artificial, with the symmetrodont molar pattern representing a structural grade that evolved multiple times^9^,^10^,^11^. In fact, weakly-triangulated molariforms, as represented by tinodontids, are present near the base of Mammalia in recent analyses^12^,^13^,^14^, suggesting that this pattern was shared by the ancestors of dentally specialised but structurally disparate groups such as eutriconodontans, multituberculates, dryolestoids, and therians. Classic studies of molar evolution have proposed homologies of cusps between therians (the group containing marsupials and placentals) and non-therians^15^,^16^,^17^, and there has been broad agreement when it comes to transformations from a symmetrodont pattern (though notable exceptions include the fate of upper molar cusp C with regards to the metacone, and the homology of the distal lower molar cusp d with the cusps of the therian talonid); see recent review by Davis^18^. Therefore, discoveries of well-preserved symmetrodont specimens have the potential to illuminate the morphological transitions that gave rise to therians.

Symmetrodont taxa with acutely-triangulated molar cusps (“acute-angled symmetrodonts”) form a monophyletic group in recent analyses^19^,^20^,^21^, positioned at the base of the Trechnotheria, a clade which also contains dryolestoids and therians. These symmetrodonts are known principally from the Early Cretaceous and became extinct prior to the Campanian^22^. They segregate into two lineages: the Spalacotheriidae, a diverse group which contain some dentally specialised taxa bearing a high number of mesiodistally compressed molars, and the Zhangheotheriidae, a group restricted to Asia and bearing a more plesiomorphic dentition. Of this latter group, only three genera have been previously described. *Zhangheotherium quinquecuspidens*^23^ is known by a nearly complete skeleton and a second, badly crushed but ontogenetically younger specimen^24^, showing evidence of tooth replacement. A second genus, *Maotherium*, is known by two specimens from the Lower Cretaceous Yixian Formation of Liaoning Province, China (the same unit that yielded *Zhangheotherium*). The type species, *M. sinensis*^25^, is represented by a flattened but fully articulated skeleton with associated soft tissue impressions. A second species, *M. asiaticus*^26^, is represented by a beautifully preserved but only partially described skeleton. Only one zhangheotheriid is known from outside China—*Kiyatherium cardiodens*^27^, from the Lower Cretaceous Ilek Formation of western Siberia. The holotype is an isolated maxilla fragment bearing the canine and most of the postcanine dentition. Additional material was described by Lopatin *et al.*^28^, including a second maxilla and several dentary fragments, permitting description of the complete lower dentition.

We describe a new zhangheotheriid, also from the Yixian Formation, represented by a partial skull and associated dentaries. Upon analysis, this taxon is positioned at the base of the Zhangheotheriidae. The rostrum and much of the orbital mosaic are well preserved. Despite the lack of preservation of the basicranium and postcranial skeleton, this specimen is the first symmetrodont in which the crown morphology for the complete dental series is fully exposed, showing a formula of I4/3 C1/1 P5/4 M3/4; as most of the record of trechnotherian diversity consists of isolated teeth and fragmentary jaws, our new specimen provides an opportunity to explore postcanine positional homology across symmetrodonts. A postcanine count of five premolars and three molars is generally regarded as primitive for therians^29^, suggesting that this tooth count may be primitive for Trechnotheria as a whole.

## Results

### Systematic Paleontology

Class Mammalia Linnaeus, 1758

Superlegion Trechnotheria McKenna^29^

Family Zhangheotheriidae Rougier, Ji, and Novacek^25^

*Anebodon luoi*, gen. et sp. nov.

**Figure 1:**
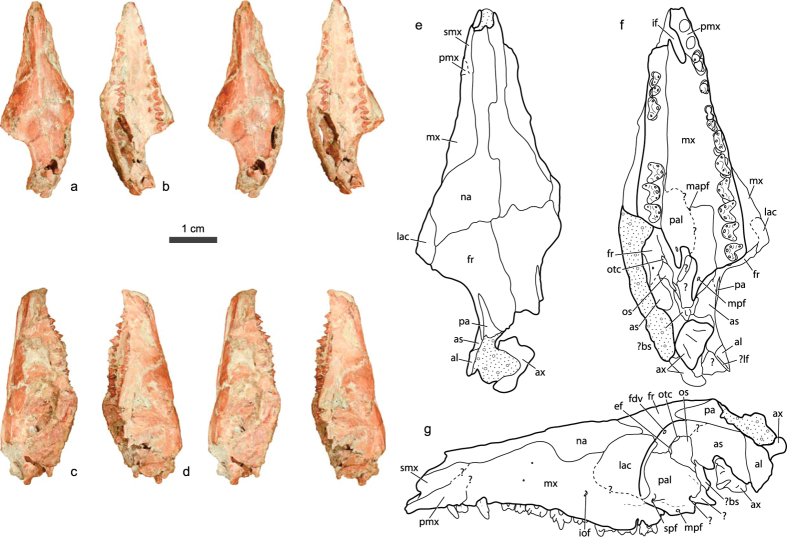
Stereophotographs of the skull of the zhangheotheriid *Anebodon luoi*, gen. et sp. nov., in dorsal (**a**), ventral (**b**), right lateral (**c**) and left lateral (**d**) views; illustrations of the skull in dorsal (**e**), ventral (**f**), and left lateral (**g**) views. Illustrations are enlarged to show detail and are not to same scale as photographs. Dotted fill represents matrix. Abbreviations: al, anterior lamina of petrosal; as, alisphenoid; ax, axis; bs, basisphenoid; ef, ethmoidal foramen; fdv, foramen for frontal diploic vein; fr, frontal; if, incisive foramen; iof, infraorbital foramen; lac, lacrimal; lf, lateral flange of petrosal; mapf, major palatine foramen; mpf, minor palatine foramen; mx, maxilla; na, nasal; os, orbitosphenoid; otc, anterior opening of orbitotemporal canal; pa, parietal; pal, palatine; pmx, premaxilla; smx, septomaxilla; spf, sphenopalatine foramen.

**Figure 2:**
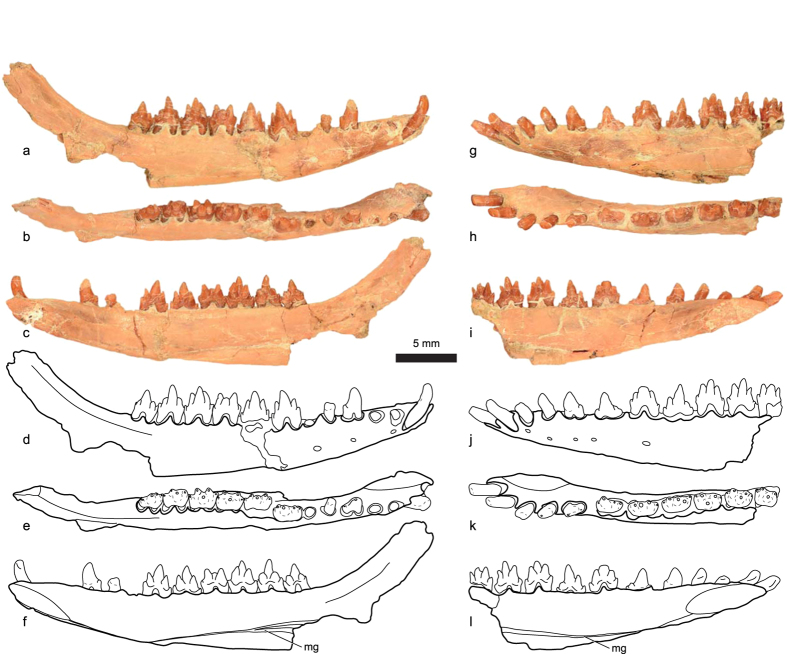
Right (**a–f**) and left (**g–l**) dentaries of the zhangheotheriid *Anebodon luoi*, gen. et sp. nov., in lateral (**a,d,g,j**), dorsal (**b,e,h,k**), and medial (**c,f,i,l**) views. Abbreviation: mg, Meckel’s groove.

**Figure 3:**
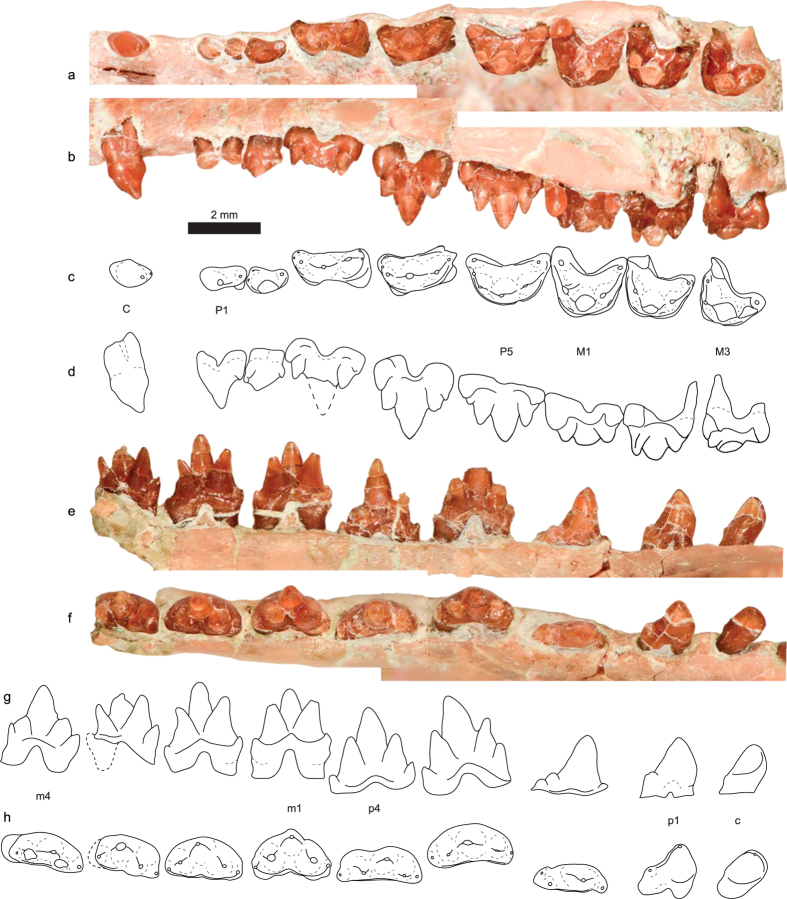
Left upper (**a–d**) and left lower (**e–h**) dentition of the zhangheotheriid *Anebodon luoi*, gen. et sp. nov., in occlusal (**a,c,f,h**), buccal (**b,d**) and lingual(**e,g**) views, showing canine and postcanine teeth. The lower m4 is reflected from the right side.

Holotype: A partial skull and associated dentaries with nearly complete upper and lower dentition (STM 38-4, Tianyu Museum of Nature, Shandong Province, China. Figs 1, 2, 3, Figs S1 and 2 and Table 1).

Etymology: From the Greek *anebos*, young, in reference to the relatively late replacement of the last premolar position, and the Greek *odontos*, tooth, a common suffix for Mesozoic mammals. The species name honours Zhe-Xi Luo for his contribution to the study of Mesozoic mammals.

Horizon and Locality: Yixian Formation, Lower Cretaceous, dated about 123 Mya; Beipiao, Liaoning Province, China.

Diagnosis: Zhangheotheriid differing from *Maotherium* and *Zhangheotherium* in having a postcanine dental formula of P5/4 M3/4, and in more obtuse triangulation of distal molars. Further differs from *Zhangheotherium* in having upper molars with deeper ectoflexus, subequal cusp B and metacone, and well-developed lingual cingulum, and lower molars with paraconid taller than metaconid. Differs from *Maotherium* and *Kiyatherium* in the absence of upper molar parastyle and less reduction of ultimate molar. Most closely resembles *Kiyatherium* but differs in having double-rooted upper canine, molariform P3/p3, and weaker upper molar ectocingulum and lingual cingulum.

### Description and Comparisons

#### Skull

A detailed description and additional images of the skull, dentary, and dentition of *Anebodon* can be found in the Supplementary Information. *Anebodon* is now the third zhangheotheriid known by cranial material. Unfortunately, most of the descriptions of *Maotherium* and *Zhangheotherium* are focused on the character-rich petrosal, which is not preserved in our new taxon. However, some comparisons with these taxa can be made from the limited overlapping described skull regions and from gleaning morphological information from the character dataset of Ji *et al.*^26^. The skull of *Anebodon* is relatively low-vaulted and generally primitive in construction (Fig. 1), as in other zhangheotheriids. While generally well preserved in three dimensions, the skull is distorted such that the right side is sheared anteriorly relative to the left side (most apparent along the posterior margin of the palate, Fig. 1b,f). This distortion affects to some degree the appearance and relative sizes of the bones in the orbitotemporal region and what remains of the sidewall of the braincase, and the associated crushing has made some sutural boundaries difficult to interpret. The slender appearance of the snout (in dorsal and ventral views) is due to the collapse of most of the right half of the palate. In general proportions, the skull of *Anebodon* would likely have resembled that reconstructed for *Maotherium sinensis*^25^.

The lateral margin of the nasal cavity is formed by a relatively large septomaxilla (Fig. 1d,g), a primitive condition shared with other zhangheotheriids. This bone bears a medially-directed horizontal process, though it is unclear if a septomaxillary foramen is present due to matrix infill. The vertical portion of the bone tapers posterodorsally but is damaged, making it impossible to determine if it contacted the maxilla (as in *Vincelestes*^30^) or if the facial process of the premaxilla separated it from the maxilla (as in docodonts^31^). The premaxilla is similarly damaged, so contact between it and the nasals cannot be determined. A moderate-sized infraorbital foramen is present on the facial portion of the maxilla above the ultimate premolar, but this region is damaged and no additional foramina are visible. The nasals are anteriorly narrow in dorsal view and broaden considerably posteriorly towards their relatively straight contact with the frontals (Fig. 1a,e), as in *Maotherium sinensis*^25^. It should be noted that the nasals are scored as posteriorly narrow by Ji *et al.*^26^ in both *Maotherium* and *Zhangheotherium*. No nasal foramina are visible.

The hard palate extends posterior to the last upper molar, and palatal vacuities and postpalatine torus are lacking (Fig. 1b,f). Alveoli for four subequal incisors, I1–I4, are contained entirely within the premaxilla. The I4 alveolus is concave laterally, contouring a pronounced groove on the root of the tooth. The canine alveolus is entirely within the maxilla. A thickened posterior prong of the palatine bears a well-defined minor palatine foramen opening into the floor of the orbit (Fig. 1f,g).

The frontal and lacrimal have limited orbital exposure, with most of the anteromedial wall of the orbit formed by a large perpendicular process of the palatine (Figs 1 and S1), the condition in mammaliaforms such as *Morganucodon* and *Haldanodon*^31^,^32^, and differing from that in multituberculates which have large orbital contributions from the frontal and maxilla^33^. While not described, the zhangheotheriids *Maotherium* and *Zhangheotherium* were scored as having expansive orbital exposure of the maxilla^26^. The ethmoidal foramen is positioned at the suture between frontal and palatine, and the frontal forms the entire anterior opening of the orbitotemporal canal (Fig. S1). A well-defined, anterodorsally-directed foramen is present at the presumed posterior extent of the palatine where this bone would have met the sphenoid complex. A possible identification for this foramen is the transverse canal, a feature common among extant metatherians and in some eutherians^34^,^35^, but so far limited among non-therians to the multituberculate *Kryptobaatar*^33^. The orbitosphenoid is comparable in development to *Morganucodon*^31^ and *Kryptobaatar*. The alisphenoid is interpreted as a relatively large and broad element, contacting the frontal at its anterodorsal margin. Much of the inferior portion of the alisphenoid is missing; accordingly, other major openings in the sidewall of the braincase cannot be identified. A thickened vertical ridge is interpreted as the suture between alisphenoid and anterior lamina of the petrosal. The posterior extent of the latter element is missing, but it appears likely that the relative proportions of these two bones were similar to the condition in the zatherian *Vincelestes*^36^.

The alveolar portions of both dentaries are well preserved, but each is missing most or all of the posteroventral portion behind the tooth row. From available evidence, the dentary of *Anebodon* differs little from other zhangheotheriids; the coronoid process ascends at a low angle (~150°), and a well-defined Meckel’s groove is present on the medial side of the dentary (Fig. 2).

#### Dentition

The dental formula of *Anebodon* is interpreted as I4/3 C1/1 P5/4 M3/4, with the left side of the specimen preserving evidence of the entire upper and lower dentition (Figs 1,2 and S2). *Anebodon* is referred to the Zhangheotheriidae based primarily on features of the dentition. *Anebodon* can be distinguished from tinodontids in that the upper and lower molars are better triangulated (Fig. 3). It differs from spalacotheriids in the strong development of cusp B and retention of a large metacone, as well as the presence of a strong lingual cingulum and the lack of mesiodistal compression of the molars. Both upper and lower canines are small in *Anebodon*, a feature shared among tinodontids and zhangheotheriids. The upper canine crown is premolariform and double-rooted, while the lower crown is incisiform and single-rooted.

The first two upper and lower premolars in *Anebodon* are premolariform. The P2 is very small and has a single, heavy root though it is grooved buccally and may divide deeper in the alveolus (Fig. 3b,d). Due to its small size, it is possible that this tooth could be interpreted as a retained DP1, but based on the very limited evidence for replacement at this position in Mesozoic mammals we prefer a conservative approach. The lower p2 bears a distinct but damaged metaconid. The remaining premolars are strongly molariform, with the upper premolars having a progressively better-developed lingual cingulum (complete on the P5). The P4 is the tallest tooth in the postcanine series, even accounting for the incomplete eruption of the P5. The crowns of the P5/p4 lack wear and are not fully erupted, as the distal base of the crowns has yet to clear the alveolar margin (Fig. 3b,e); evidence of replacement at these tooth positions establishes a minimum upper and lower premolar count which we interpret to be the full adult condition (see Discussion below). Among zhangheotheriids, *Anebodon* differs most strikingly from the well-represented *Zhangheotherium* and *Maotherium* in postcanine dental formula. These two genera, known by multiple nearly complete skeletons, have postcanine counts of P2/3 M5/5-6 and P1-2/1-3 M4-5/6, respectively^23^,^24^,^25^,^26^. These differ considerably from the count of P5/4 M3/4 in *Anebodon*. We consider the increase in number of molars in some zhangheotheriids to be derived (but see discussion below), with our new taxon more closely resembling tinodontids in this regard.

The mesial molars in *Anebodon* are more acutely triangulated than the ultimate premolars (M1 = 95°, m1 = 85°). The main accessory cusps are well developed, though the metacone/metaconid and metastylar region decrease in size distally (Figs 3 and S2). The upper molars have a complete and rather crenulated lingual cingulum. Molars of *Anebodon* differ from *Zhangheotherium* and *Maotherium* in lacking progressively more acute triangulation of the three main cusps distally through the molar series, a feature which these other taxa share with more derived spalacotheriids^22^. Instead, the cusp angle in *Anebodon* increases slightly distally, and by the ultimate lower molar (m4) the main cusps are nearly in-line (Figs 3h and S2k). *Anebodon* further differs from *Zhangheotherium* in the strong development of the upper molar lingual cingulid and greater depth of the ectoflexus; upper molars of the new taxon differ from those of *Maotherium* in lacking a parastyle and development of a mesially-projecting parastylar hook.

Among known zhangheotheriids, comparisons to *Kiyatherium cardiodens*^28^ are most favourable. The two partial maxillae referred to *Kiyatherium* preserve evidence of the entire postcanine dentition, originally interpreted as P1–4 and M1–4 with the first upper molar undergoing replacement (designated as RM1^28^). However, molars are by definition never replaced^37^, and this fifth postcanine should be identified as the P5. Contrary to the original interpretation, it is likely that both maxillae referred to *Kiyatherium* bear the entire molar series, with the M3 more reduced in size in the holotype specimen^28^. Though damaged, it is clear that the P5 of the referred specimen is less worn than the M1 and belongs to a different tooth generation. Under our interpretation, *Anebodon* and *Kiyatherium* share a postcanine dental formula of P5/4 M3/4. These two taxa are also broadly similar in molar morphology, and differ only in that *Anebodon* possesses a relative more molariform the third premolar, double-rooted upper canine, weaker development of upper molar cingula, and less reduction of the ultimate upper and lower molars.

## Discussion

Zhangheotheriids are trechnotherian mammals forming the sister group to the Spalacotheriidae in most recent analyses^13^,^19^,^38^. Despite limited taxonomic diversity, zhangheotheriids are known by relatively well-preserved material including partial skulls and nearly complete skeletons^23^,^24^,^25^,^26^,^28^. The known record of this group is restricted to the Early Cretaceous of China and Siberia. Zhangheotheriids share a peculiar morphology of the posterior part of the dentary, namely a deep notch separating the slender, reclined coronoid process from the stout, upturned condylar process. As in spalacotheriids, an angular process is absent but a medially-directed pterygoid flange is generally developed along the ventral and posterior margin of the postdentary region. The molariforms in zhangheotheriids are somewhat intermediate in morphology between tinodontids and spalacotheriids; the primary cusps are somewhat more acutely triangulated than in tinodontids and further decrease in angle posteriorly through the molar series, especially with regards to the upper molars (though the angle actually increases distally in *Anebodon* and *Kiyatherium*, Fig. 3). The upper molars are further derived (with respect to tinodontids) in the tall height of cusp B as well as the possession of a strong lingual cingulum. However, the molar cusps are conical and lack tight, preformed occlusal fit with the opposing teeth, which develops only after wear (a primitive condition). The apices of the stylocone and metastyle only participate in shearing once the pre- and postvallum crests have worn considerably.

**Figure 4:**
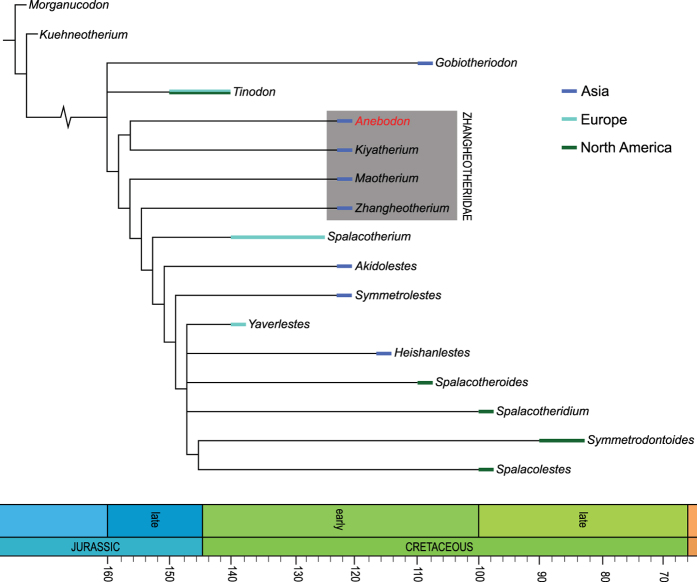
Cladogram of relationships of selected zhangheotheriid and spalacotheriid trechnotherian mammals, modified from the analysis of Sweetman^51^. Colours represent global distribution of taxa. Taxa in grey box represent the Zhangheotheriidae, a paraphyletic group in this analysis. Time scale modified from Gradstein *et al.*^53^

Most of these dental and mandibular features uniting zhangheotheriids are either plesiomorphic or can be viewed as transitional between tinodontids and more derived spalacotheriids. Not surprisingly, our result suggests that Zhangheotheridae represent a paraphyletic group, with *Anebodon* and *Kiyatherium* occupying a basal position (Fig. 4). These two taxa are least derived among zhangheotheriids with respect to distal molar triangulation and number of upper molars. However, it is worth noting that *Anebodon* and *Kiyatherium* have a postcanine dental formula of P5/4 M3/4, a condition shared (at least in part) with basal therians (the aegialodontid *Kielantherium* likely has a lower postcanine count of p4/m4^39^). In contrast, spalacotheriids increase the number of molariforms up to seven. This suggests that there were two evolutionary trends among acute-angled symmetrodonts: one retaining a relatively low molar count leading to therians, and the other increasing molar count to specialised spalacotheriids (and occurring in parallel among dryolestoids^22^).

An additional feature which appears to characterise zhangheotheriids is the strong molarization of the posterior premolars. Moreover, based on evidence from *Anebodon*, the ultimate premolar is replaced at a relatively advanced ontogenetic stage; instead of occurring in concert with eruption of the last molar, as is characteristic of therians^40^, the ultimate premolar was replaced after the last molar had accumulated substantial wear (Figs 3 and S2a). The lack of a clear morphological break in the postcanine series has led to differences in nomenclature with regards to the premolar-molar boundary in this and similar groups, with related inconsistencies in tooth formulae. For instance, specimens of the zhangheotheriid *Kiyatherium* from the Early Cretaceous of Siberia record clear replacement of the fifth postcanine upper tooth (a molariform), yet this position is identified as the M1 with the successional tooth given the awkward moniker of ‘RM1’ for ‘replacement M1’^41^. Our specimen has the same number of postcanine teeth and shows evidence of replacement at the same locus (Fig. 3). Molars, as defined by Owen^37^, are a posterior extension of the primary dental series (derived from the primary dental lamina) and are not replaced. Premolars, on the other hand, are defined as postcanines which exhibit replacement; as such, deciduous premolars belong to the primary dental series and adult premolars to the secondary series. In some instances, anterior postcanines are not replaced (e.g., P1 in eutherians); the definition can simply be amended such that a molar is any tooth posterior to the last postcanine showing evidence of replacement. Accordingly, we regard the tooth in question in *Anebodon* and *Kiyatherium* as the ultimate premolar (P5). Along these lines, it is possible that the postcanine formula in other zhangheotheriids should be reinterpreted. In the juvenile specimen of *Zhangheotherium*^26^, the tooth identified as the m1 may instead be the dp4. The p3 position is undergoing replacement—if premolar replacement was alternating, as in *Dryolestes* and basal therians^42^,^43^ and as appears to be the case in *Zhangheotherium*, the presence of an unshed dp2 implies that the fourth postcanine is the dp4 and that this tooth will likely be replaced at a later ontogenetic stage. This interpretation matches the relatively early ontogenetic age of this individual (relative to the holotype of *Zhangheotherium quinquecuspidens*), and reduces the disparity in lower dental formula among zhangheotheriids.

Far from an issue of semantics, identification of postcanine tooth positions affects interpretation of homology across Mesozoic lineages (particularly those at the base of the therian line) and reconstruction of ontogenetic processes such as the timing and pattern of tooth replacement. We interpret our specimen to have an upper postcanine formula of P5/M3, which is generally thought to be the primitive count for therians^29^,^44^. Additionally, the penultimate upper premolar (P4) in *Anebodon* is the tallest tooth in the postcanine series, a feature also characteristic of therians^29^. Some degree of molarization of the posterior premolars also occurs in various spalacotheriids^45^,^46^,^47^, but evidence of replacement of strongly molariform teeth is currently lacking in this derived group. Retention of molariform premolars in the adult dentition is also a characteristic of the endemic South American meridiolestidan dryolestoids^48^; the distribution of this feature suggests that it is basal to the Trechnotheria and possibly represents the primitive condition for the therian line^25^. Ultimately, the craniodental morphology of *Anebodon* and the implications raised by its placement at the base of a paraphyletic Zhangheotheriidae highlight the need for a more expansive analysis to explore the interrelationships of basal trechnotherians and, more pointedly, the role these symmetrodont taxa play in an improved understanding of therian evolution.

## Methods

### Terminology and conventions

Dental measurements are shown in Table 1. We follow the measurement conventions and terminologies of Cifelli and Madsen^49^ and Rougier *et al.*^25^,^50^. However, we employ traditional therian terminology for tooth structures that have been proposed as homologous in symmetrodonts (e.g., cusp C = metacone, cusp d = hypoconid); see recent review in Davis^18^.

### Phylogenetic analysis

The data matrix was based on that of Sweetman^51^ with an additional character (molarization of the ultimate premolar; see the Supplementary Information for complete character list). Our new taxon and *Kiyatherium* were added to the matrix. In addition, we updated some scores for *Maotherium* for characters 3, 24, and 28 based on our observations (BD). The data matrix consisting of 17 taxa and 30 characters was analyzed using a traditional search of TNT^52^. All characters are equally-weighted and non-additive. The TNT analysis produced 10 most parsimonious trees with a tree length of 65 steps, a consistency index of 0.53, and a retention index of 0.72; a strict consensus of these is presented in Fig. 4.

## Additional Information

**How to cite this article**: Bi, S. *et al.* A new symmetrodont mammal (Trechnotheria: Zhangheotheriidae) from the Early Cretaceous of China and trechnotherian character evolution. *Sci. Rep.* **6**, 26668; doi: 10.1038/srep26668 (2016).

## Supplementary Material

Supplementary Information

## Figures and Tables

**Table 1:** Measurements (in mm) of the dentition of the zhangheotheriid *Anebodon luoi*, gen. et sp. nov. L-left side; R-right side.

Upper Teeth	Length-L	Width-L	Length-R	Width-R	Lower Teeth	Length-L	Width-L	Length-R	Width-R
I1*	1.31	0.95			i1	0.81	0.4		
I2*	1.29	0.88			i2	0.78	0.38	0.8	0.41
I3	0.89	0.72			i3	0.78	0.51		
I4	1.02	0.81							
C	1.03	0.7			c	0.9	0.77		
P1			1.38	0.65	p1	1.51	0.8	1.5	0.87
P2	1.12	0.71	0.98	0.7	p2	2.12	1.08		
P3	2.35	1.02	2.4	1.01	p3	2.3	1.11	2.31	1.03
P4	2.4	1.15			p4	2.45	1.13	2.33	
P5	2.55	1.5			m1	2.26	1.28	2.29	1.29
M1	2.32	1.99	2.25	2.0	m2	2.28	1.31	2.3	1.33
M2	2.1	1.88	2.13	1.9	m3	2.02	1.2	2.1	1.21
M3	2.05	1.75	2.09	1.79	m4			1.85	0.99

Note: *I1 and I2 are not preserved and the measurements were taken from the alveoli.

## References

[b1] Simpson G. G. Mesozoic Mammalia. III. Preliminary comparison of Jurassic mammals except multituberculates. Am. J. Sci. 10, 559–569 (1925).

[b2] Simpson G. G. A Catalogue of the Mesozoic Mammalia in the Geological Department of the British Museum. (Trustees of the British Museum, 1928).

[b3] Simpson G. G. American Mesozoic Mammalia. Memoirs of the Peabody Museum 3, 1–235 (1929).

[b4] Kermack D. M., Kermack K. A. & Mussett F. The Welsh pantothere *Kuehneotherium praecursoris*. Zool. J. Linn.Soc. 47, 407–423 (1968).

[b5] Chow M. & Rich T. H. *Shuotherium dongi*, n. gen. and sp., a therian with pseudo-tribosphenic molars from the Jurassic of Sichuan, China. Aust. Mammal. 5, 127–142 (1982).

[b6] Sigogneau-Russell D. Discovery of a Late Jurassic Chinese mammal in the upper Bathonian of England. Comptes Rendus de l’Académie des Sciences, Paris 327, 571–576 (1998).

[b7] Cassiliano M. L. & Clemens W. A. In Mesozoic Mammals: The First Two-thirds of Mammalian History (eds Lillegraven J. A., Kielan-Jaworowska Z. & Clemens W. A. ) 150–161 (University of California Press, 1979).

[b8] McKenna M. C. & Bell S. K. Classification of Mammals Above the Species Level. (Columbia University Press, 1997).

[b9] Rowe T. B. Definition, diagnosis, and origin of Mammalia. J. Vert. Paleontol. 8, 241–264 (1988).

[b10] Rougier G. W., Wible J. R. & Hopson J. A. Basicranial anatomy of *Priacodon fruitaensis* (Triconodontidae, Mammalia) from the Late Jurassic of Colorado, and a reappraisal of mammaliaform interrelationships. Am. Mus. Novit. 3183, 1–38 (1996).

[b11] Luo Z.-X., Kielan-Jaworowska Z. & Cifelli R. L. In quest for a phylogeny of Mesozoic mammals. Acta Palaeontol. Pol. 47, 1–78 (2002).

[b12] Luo Z.-X. *et al.* Evolutionary development in basal mammaliaforms as revealed by a docodontan. Science 347, 760–764, 10.1126/science.1260880 (2015). 25678660

[b13] Rougier G. W., Wible J. R., Beck R. & Apesteguía S. The Miocene mammal *Necrolestes* demonstrates the survival of a Mesozoic non-therian lineage into the late Cenozoic of South America. Proc. Natl Acad. Sci. USA 109, 20053–20058 (2012). 23169652 10.1073/pnas.1212997109 PMC3523863

[b14] Martin T. *et al.* A Cretaceous eutriconodont and integument evolution in early mammals. Nature 526, 380–384, 10.1038/nature14905 (2015). 26469049

[b15] Butler P. M. The teeth of the Jurassic mammals. Proc. Zool. Soc. Lond. 109, 329–356 (1939).

[b16] Patterson B. Early Cretaceous mammals and the evolution of mammalian molar teeth. Fieldiana: Geology 13, 1–105 (1956).

[b17] Crompton A. W. In Early Mammals (eds Kermack D. M. & Kermack K. A. ) 65–87 (Zool. J. Linn. Soc., Suppl. 1, London, 1971).

[b18] Davis B. M. Evolution of the tribosphenic molar pattern in early mammals, with comments on the “Dual-Origin” hypothesis. J. Mamm. Evol. 18, 227–244 (2011).

[b19] Krause D. W. *et al.* First cranial remains of a gondwanatherian mammal reveal remarkable mosaicism. Nature 515, 512–517, 10.1038/nature13922 (2014). 25383528

[b20] Luo Z.-X., Gatesy S. M., Jenkins F. A., Amaral W. W. & Shubin N. H. Mandibular and dental characteristics of Late Triassic mammaliaform *Haramiyavia* and their ramifications for basal mammal evolution. Proc. Natl Acad. Sci. USA 112, E7101–E7109 10.1073/pnas.1519387112 (2015). 26630008 PMC4697399

[b21] Bi S., Wang Y., Guan J., Sheng X. & Meng J. Three new Jurassic euharamiyidan species reinforce early divergence of mammals. Nature 514, 579–584 10.1038/nature13718 (2014). 25209669

[b22] Kielan-Jaworowska Z., Cifelli R. L. & Luo Z.-X. Mammals from the Age of Dinosaurs: Structure, Relationships, and Paleobiology. (Columbia Univeristy Press, 2004).

[b23] Hu Y.-M., Wang Y.-Q., Luo Z.-X. & Li C.-K. A new symmetrodont mammal from China and its implications for mammalian evolution. Nature 390, 137–142 (1997). 9367151 10.1038/36505

[b24] Luo Z.-X. & Ji Q. New study on dental and skeletal features of the Cretaceous “symmetrodontan” mammal *Zhangheotherium*. J. Mamm. Evol. 12, 337–357 (2005).

[b25] Rougier G. W., Ji Q. & Novacek M. J. A new symmetrodont mammal with fur impressions from the Mesozoic of China. Acta Geol. Sin.(Engl.) 77, 7–14 (2003).

[b26] Ji Q., Luo Z.-X., Zhang X., Yuan C.-X. & Xu L. Evolutionary development of the middle ear in Mesozoic therian mammals. Science 326, 278–281 (2009). 19815774 10.1126/science.1178501

[b27] Maschenko E. N., Lopatin A. V. & Voronkevich A. V. A new Early Cretaceous mammal from western Siberia. Dokl. Biol. Sci. 386, 715–717 (2002). 10.1023/a:1020791007455 12469418

[b28] Lopatin A. V., Averianov A. O., Maschenko E. N. & Leshchinskiy S. V. Early Cretaceous mammals of Western Siberia: 3. Zhangheotheriidae. Paleontological Journal 44, 573–583 (2010).

[b29] McKenna M. C. In Phylogeny of the Primates (eds Luckett W. P. & Szalay F. S. ) 21–46 (Plenum Press, 1975).

[b30] Rougier G. W. Vincelestes neuquenianus Bonaparte (Mammalia, Theria) un Primitivo Mamífero del Cretácico Inferior de la Cuenca Neuquina, Universidad Nacional de Buenos Aires (1993).

[b31] Kermack K. A., Mussett F. & Rigney H. W. The skull of *Morganucodon*. Zool. J. Linn. Soc. 71, 1–158 (1981).

[b32] Lillegraven J. A. & Krusat G. Cranio-mandibular anatomy of *Haldanodon exspectatus* (Docodonta; Mammalia) from the Late Jurassic of Portugal and its implications to the evolution of mammalian characters. Contrib. Geol. Univ. Wyo. 28, 39–138 (1991).

[b33] Wible J. R. & Rougier G. W. The cranial anatomy of *Kryptobaatar dashzevegi* (Mammalia, Multituberculata), and its bearing on the evolution of mammalian characters. Bull. Am. Mus. Nat. Hist. 247, 1–124 (2000).

[b34] MacPhee R. D. E. Morphology, adaptations, and relationships of *Plesiorycteropus*: and a diagnosis of a new order of eutherian mammals. Bull. Am. Mus. Nat. Hist. 220, 1–214 (1994).

[b35] Sánchez-Villagra M. R. & Wible J. R. Patterns of evolutionary transformation in the petrosal bone and some basicranial features in marsupial mammals, with special reference to didelphids. J. Zool. Syst. Evol. Res. 40, 26–45, 10.1046/j.1439-0469.2002.00173.x (2002).

[b36] Hopson J. A. & Rougier G. W. Braincase structure in the oldest known skull of a therian mammal: implications for mammalian systematics and cranial evolution. Am. J. Sci. 293, 268–299 (1993).

[b37] Owen R. On the Anatomy of Vertebrates. Volume III. Mammals. (Longmans, Greens and Co., 1868).

[b38] Zhou C.-F., Wu S., Martin T. & Luo Z.-X. A Jurassic mammaliaform and the earliest mammalian evolutionary adaptations. Nature 500, 163–167, 10.1038/nature12429 (2013). 23925238

[b39] Dashzeveg D. & Kielan-Jaworowska Z. The lower jaw of an aegialodontid mammal from the Early Cretaceous of Mongolia. Zool. J. Linn. Soc. 82, 217–227 (1984).

[b40] Luckett W. P. In Mammal Phylogeny, Volume 2–Mesozoic Differentiation, Multituberculates, Monotremes, Early Therians, and Marsupials Vol. 1 (eds Sigmond Szalay Frederick, Novacek Michael J. & McKenna Malcolm C. ) 182–204 (Springer-Verlag, Inc., 1993).

[b41] Lopatin A. V. *et al.* Early Cretaceous mammals from western Siberia. 1. Tinodontidae. Paleontologiceskij Zurnal 39, 523–534 (2005).

[b42] Martin T. Tooth replacement in Late Jurassic Dryolestidae (Eupantotheria, Mammalia). J. Mamm. Evol. 4, 1–18 (1997).

[b43] Davis B. M. A novel interpretation of the tribosphenidan mammal *Slaughteria eruptens* from the Early Cretaceous Trinity Group, and implications for dental formula in early mammals. J. Vert. Paleontol. 31, 676–683 (2011).

[b44] O’Leary M. A. *et al.* The placental mammal ancestor and the post–K-Pg radiation of placentals. Science 339, 662–667, 10.1126/science.1229237 (2013). 23393258

[b45] Tsubamato T., Rougier G. W., Isaji S., Manabe M. & Forasiepi A. M. New Early Cretaceous spalacotheriid “symmetrodont” mammal from Japan. Acta Palaeontol. Pol. 49, 329–346 (2004).

[b46] Cifelli R. L. Therian teeth of unusual design from the medial Cretaceous (Albian-Cenomanian) Cedar Mountain Formation, Utah. J. Mamm. Evol. 6, 247–270 (1999).

[b47] Hu Y.-M., Fox R. C., Wang Y. & Li C.-K. A new spalacotheriid symmentrodont from the Early Cretaceous of northeastern China. Am. Mus. Novit. 3475, 1–20 (2005).

[b48] Rougier G. W., Apesteguía S. & Gaetano L. Highly specialized mammalian skulls from the Late Cretaceous of South America. Nature 479, 98–102 (2011). 22051679 10.1038/nature10591

[b49] Cifelli R. L. & Madsen S. K. Spalacotheriid symmetrodonts (Mammalia) from the medial Cretaceous (upper Albian or lower Cenomanian) Mussentuchit local fauna, Cedar Mountain Formation, Utah, USA. Geodiversitas 21, 167–214 (1999).

[b50] Rougier G. W., Spurlin B. K. & Kik P. K. A new specimen of *Eurylambda aequicrurius* and considerations on “symmetrodont” dentition and relationships. Am. Mus. Novit. 3394, 1–15 (2003).

[b51] Sweetman S. C. A spalacolestine spalacotheriid (Mammalia, Trechnotheria) from the Early Cretaceous (Barremian) of southern England and its bearing on spalacotheriid evolution. Palaeontology 51, 1367–1385 (2008).

[b52] Goloboff P. A. Analyzing large data sets in reasonable times: solutions for composite optima. Cladistics 15, 415–428 (1999). 10.1111/j.1096-0031.1999.tb00278.x 34902941

[b53] Gradstein F. M., Ogg J. G., Schmitz M. D. & Ogg G. M. The Geologic Time Scale 2012. (Elsevier, 2012).

