# Supplementary material for: A new symmetrodont mammal (Trechnotheria: Zhangheotheriidae) from the Early Cretaceous of China and trechnotherian character evolution
Source: Sci Rep. 2016 May 24;6:26668. doi: 10.1038/srep26668 (PMC4877676; doi:10.1038/srep26668)
Supplement: Supplementary Information [file srep26668-s1.pdf]

A new symmetrodont mammal (Trechnotheria: Zhangheotheriidae) from the Early Cretaceous of China and trechnotherian character evolution

Shundong Bi[1,2]*, Xiaoting Zheng[3,4], Jin Meng[5,2], Xiaoli Wang[3,4], Nicole Robinson[1], and Brian Davis[6*]

[1]Department of Biology, Indiana University of Pennsylvania, Indiana, PA 15705, USA.

[2]Key Laboratory of Vertebrate Evolution and Human Origins of Chinese Academy of Sciences, Institute of Vertebrate Paleontology and Paleoanthropology, Chinese Academy of Sciences, Beijing 100044.

[3] Shandong Tianyu Museum of Nature, Pingyi, Shandong, China.

[4] Institute of Geology and Paleontology, Linyi University, Linyi, Shandong, China

[5] Division of Paleontology, American Museum of Natural History, Central Park West at 79th St., New York, NY 10024, USA.

[6] Department of Anatomical Sciences and Neurobiology, University of Louisville, 511 S. Floyd St., Louisville, KY 40202, USA. bm.davis@louisville.edu

[*]Corresponding author

Supplementary Information

Here we provide a full, detailed description of the skull and dentition of *Anebodon luoi*, gen. et sp. nov., as well as additional figures of the specimen. We also provide a list of characters for the modified analysis of Sweetman (2008)[1].

Full description

Skull.—The nasal cavity is somewhat collapsed and is still filled with matrix, but the margins of the nares are mostly preserved on the left side. The lateral margin is formed by a relatively large septomaxilla, a primitive condition shared with other zhangheotheriids. This bone bears a medially-directed horizontal process, but it is unclear if a septomaxillary foramen is present due to matrix infill. The vertical portion of the septomaxilla tapers posterodorsally, but the posteriormost portion of the bone is missing and it is impossible to determine if it contacted the maxilla (as in *Vincelestes*[2]) or if the facial process of the premaxilla separated it from the maxilla (as in docodonts[3]). The premaxilla has a relatively small facial process, which is similarly damaged at its posterodorsal extent; thus, the boundary with the maxilla is difficult to interpret and it is uncertain if the premaxilla contacted the nasal. While the premaxilla is described as contacting the nasal in the zhangheotheriid *Maotherium sinensis*[4], contact is scored as lacking in *M. asiaticus* by Ji *et al*. (2009)[5]. The septomaxilla-premaxilla suture is oblique and ends with the premaxilla extending slightly to form the anterior-most portion of the snout. The facial process of the maxilla is tall and expansive. Based on its contact with the premaxilla in ventral view, it is likely that a lappet of the facial process of the maxilla covered some portion of the premaxilla, but this contact is damaged. A moderate-sized infraorbital foramen is present above the ultimate premolar, but bilateral damage to the posterior portion of the maxilla makes it difficult to determine if any additional infraorbital foramina were present, though breakage has exposed the infraorbital canal from its posterior opening in the orbit. The contact with the jugal cannot be described as it is too damaged, and most of the boundary with the lacrimal is difficult to interpret. The nasals in dorsal view are narrow anteriorly and transversely expanded posteriorly, and contact the frontals along a broad, relatively straight suture. No nasal foramina are visible.

The left half of the palate is relatively complete and undistorted, with the hard palate extending posterior to the ultimate molar. The premaxilla contains subequal alveoli for four incisors. The I1 and I2 are well spaced, but the I3 and I4 are crowded. The alveoli for I1–I3 are circular, while the lateral margin of the I4 alveolus is concave, contouring a pronounced groove on the root of the tooth (described with the dentition, below). Small but distinct pits are present at the posteromedial margin of the alveoli, presumably to accommodate the tips of the lower incisor crowns during full closure of the jaws. The premaxilla forms most of the anterolateral margin of a large, obliquely-oriented incisive foramen; the anteromedial-most part of this structure is missing. The palatal exposure of the premaxilla narrows in the vicinity of the I3 but is visible as a thin rim forming the entirety of the I3 and I4 alveoli. The maxilla is extensive and forms most of the hard palate. Anteriorly, it forms the posterior one-quarter and the entire preserved medial margin of the incisive foramen. The maxilla contacts the premaxilla just medial to the I3 and I4 alveoli and continues to wrap anterolaterally around the I4 alveolus before continuing dorsally as the facial process. While no large paracanine fossa is present, shallow accommodation pits for the lower teeth are present medial to the alveoli of each tooth position. No palatal vacuities are present. Distortion and crushing have forced the right palatine to ventrally cover much of the left palatine, obliterating the contact between the two bones and disturbing much of the transverse suture between the maxilla and palatine. A small concave margin of bone along the interpreted suture appears natural, and is here identified as the position of the major palatine foramen. The horizontal portion of the palatine extends anteriorly to the level of the anterior M1 alveolus. Posteriorly, this bone frames most of the choanae, the ventral margin of which is preserved but distorted. While the bone forming this posterior rim of the hard palate is somewhat thickened, a postpalatine torus does not appear to be developed. The palatine

continues posteriorly and laterally beyond the choanae as a thick prong which bears the minor palatine foramen; this foramen opens directly into the floor of the orbit. An elongate splint of bone, possibly representing a displaced portion of a pterygoid, is present at the posterior opening of the choanae.

Only the anterior portion of the skull roof is preserved. The frontals are largely complete but somewhat distorted, such that they appear asymmetrical. The bones are broad anteriorly, and along with the lacrimals form the orbital rim. The frontals narrow posteriorly where they meet a prong of the parietals just behind the orbital rim, but the dorsal suture with the parietals is not completely preserved.

The orbital exposure of the frontal is rather limited, with the perpendicular process of the palatine forming most of the anteromedial wall of the orbit. This condition differs from multituberculates[6] and more closely resembles primitive mammaliaforms such as docodonts and *Morganucodon*[3,7]. The lacrimal also has limited exposure on the anterior wall of the orbit; this bone is damaged, and a lacrimal foramen is not preserved. A small, oval foramen to accommodate the frontal diploic vein is present in the frontal just below the orbital rim. The orbital portion of the frontal extends inferiorly to participate in the ethmoidal foramen, where it meets the palatine. The frontal forms the entire anterior opening of the orbitotemporal canal and posteriorly contacts the orbitosphenoid and alisphenoid, though this latter suture is difficult to identify. Dorsally, the perpendicular process of the palatine meets the frontal and forms part of the anterior margin of the ethmoidal foramen. A deep pocket in the anteroventral corner of the orbit contains the sphenopalatine foramen, which is housed entirely within the palatine. A narrow shelf lateral to the foramen accommodated the associated neurovasculature, and grooves lead posteriorly towards the orbital opening of the minor palatine foramen, anteriorly towards the

infraorbital canal, and dorsally into the orbit. Posteriorly, the palatine has a broad contact with the orbitosphenoid, but much of the posteroventral region of the orbitotemporal region is missing. A well-defined foramen is preserved on the left side, opening posteroventrally and presumably at the posterior extent of the palatine where this bone would have met the sphenoid complex (indeterminate bone fragments are covering this region on the right side of the skull). A possible identification for this foramen is the transverse canal, which transmits the transverse canal vein communicating between the orbitotemporal region and cavernous sinus. The presence of a transverse canal is widespread among extant metatherians and is present in some eutherians[8,9], suggesting that it may be a primitive therian feature, but among non-therians it has been identified only in the multituberculate *Kryptobaatar*[6]. From their data matrix, a transverse canal is reported as absent by Ji *et al*. (2009)[5]in both *Maotherium* and *Zhangheotherium*.

The orbitosphenoid, as interpreted, is a small but broad bone exposed mainly high in the orbitotemporal region. The ethmoidal foramen is located where the orbitosphenoid meets the palatine and frontal, and a prominent cleft running along the contact between orbitosphenoid and frontal contains both this foramen and the anterior opening of the orbitotemporal canal. The orbitosphenoid tapers posteroventrally along its presumed contact with the alisphenoid, but most of the ventral sidewall of the braincase has been lost. A thickened vertically-oriented ridge towards the preserved posterior extent of the sidewall of the braincase is here interpreted as the suture between the alisphenoid and anterior lamina of the petrosal; under this interpretation, the alisphenoid is large though breakage of the anterior laminal of the petrosal precludes a determination of the relative sizes of the two elements. However, it is likely that these bones were roughly equivalent in size in *Anebodon*, more closely resembling the condition in *Vincelestes* than that in multituberculates[10]. The alisphenoid likely had a limited contact with the

frontal and met the parietal along most of its dorsal edge. Lack of preservation of the ventral half of the alisphenoid prevents identification of foramina for branches of the trigeminal nerve as well as other features such as a sphenorbital fissure or optic foramen. The area of bone here interpreted as the anterior lamina of the petrosal has a thickened ventral margin, which may represent the lateral flange. The portions of the skull dorsal and posterior to this element are missing.

All that remains of the basicranium is a wedge-shaped bone here interpreted as a possible displaced and damaged basisphenoid. It is visible laterally as a narrow piece of bone situated obliquely between the perpendicular process of the palatine and the alisphenoid; in this view, the element has little discernable morphology. Ventrally, the bone tapers and thickens to a somewhat cup-shaped articular surface. Most of the details of this element can be seen in a slightly oblique posterior view from the right side (such as presented in Supplementary Fig. S1). The thickened, rounded end of the bone widens and flattens, and a pair of ridges is present which run along much of the length of the bone. A small foramen is positioned medial to each ridge, and if this bone is indeed the basisphenoid these foramina would transmit the internal carotid arteries. Under this interpretation the thick, cup-shaped end of the bone represents the anterior portion of the basisphenoid where it would contact the presphenoid, and the bone has been folded dorsally from its position in life. The surface with the ridges and exposed internal carotid foramina would be the ventral surface of the element. Some support for breakage of the skull in this manner is provided by the presence of vertebral elements deep within the braincase. The axis, while nearly complete, was rotated almost 180° before coming to rest against the inner wall of the alisphenoid.

Dentary.—Like other zhangheotheriids, the dentary is long and slender, with an unfused, subhorizontal symphysis. On the medial side, the symphyseal scar extends posteriorly to the level of the lower canine. The dentary is thin and elongate, and achieves its maximum depth below the m3. Meckel's groove is shallow and tapers anteriorly to reach the ventral surface of the dentary below the p3 where it blends with the symphysis. It is broad posteriorly to the point where the dentary is broken below the m4. The ventral part of the ramus, including the angular region, is missing. The coronoid process rises posteriorly at a low angle of approximately 150° with the tooth row. While the tip of the coronoid process and part of posterior edge is broken, the general shape is similar to other zhangheotheriids. Laterally, there are four anterior mental foramina on the left side (below the i3, c, p1, and p2) and three on the right side. An additional larger, posteriormost mental foramen is present below the p3 on the left dentary, but this region is damaged on the right dentary. The limits of the masseteric fossa cannot be determined due to breakage, but judging from what is preserved, the masseteric fossa is poorly defined and does not extend anteriorly below the last molar (m4).

Lower dentition.—The tooth formula is interpreted as I4/3 C1/1 P5/4 M3/4. The left dentary preserves the first complete incisor series known among zhangheotheriids. All three incisors have spatulate crowns and are increasingly procumbent anteriorly, with i1 almost horizontal. The incisors are separated by small gaps. The buccal side of the crown is slightly convex but the lingual side is flat and marked by a median ridge. The i2 and i3 are slightly smaller in size but have a more prominent middle ridge on their lingual surface. The tip of the i3 is broken.

The canine is preserved only on the left side. The tooth is single rooted and incisiform, differing from the preceding incisors in morphology only in having a slightly more conical

crown. It is identified as the canine because it occludes between the I4 and upper canine. There is no diastema separating the lower canine from the first premolar.

The p1 is double-rooted and subequal to slightly taller than the canine. The crown is simple and premolariform with a small distal heel. A slightly longer gap separates the p1 from p2 than separates the p1 from the canine. The remaining premolars are progressively more molariform. The p2 was displaced on the left side and mostly broken on the right side. Judged from the right side, it was fully erupted with the base of the crown well above the alveolar level. The p2 is dominated by a tall, trenchant and mesiodistally elongated main cusp. A paraconid is absent and the metaconid broken but was likely low. There is a small cusp e at the mesial base and tiny, damaged distal heel (cusp d or hypoconid). The p3 and p4 are strongly molariform but less triangular than the molars. The p4 crown is not fully erupted, indicating that this tooth underwent replacement and supporting the identification of it and the preceding tooth as premolars. The p3 has well-developed paraconid and metaconid, forming a trigonid angle of approximately 135°. The main cusps are conical. Compared to the molars, the paraconid and metaconid are more slender and lower relative to the protoconid. The paraconid arches mesially. There is a weak but continuous lingual cingulid but no buccal cingulid. Cusp e is low and broad (chisel shaped). The hypoconid is low and positioned at the base of crown. The cusps are sharp and largely free of wear, suggesting the crown is freshly erupted. The p4 is generally similar to the p3, except that on the p4 the paraconid and metaconid are relatively more robust and the trigonid angle is more acute.

The mesial lower molars have a trigonid angle of approximately 85°, though the angle increases to 95° and 140° through the m3 and m4, respectively. The protoconid is the tallest cusp of the crown; the paraconid and metaconid are lower, with the paraconid the heavier and taller of

the two and the metaconid decreasing in height distally through the molar series. The main cusps are conical with the paraconid and metaconid well separated from the protoconid. On the m1, cusp e and the hypoconid are similarly developed as on the p4, but cusp e increases in size distally and becomes more procumbent through the molars. The hypoconid also increases in size between the m1 and m3 but is much smaller and closely appressed to the metaconid on the m4. While much smaller, the hypoconid is positioned higher on the crown than cusp e on the m4. The m1 and m2 have a well-developed lingual cingulid, but this feature is present only along the base of the metaconid on m3 and is absent entirely on m4. A buccal cingulid is absent on all of the molars. In addition to having a very obtuse trigonid angle, the m4 is smaller and lower-crowned than the other molars. Shearing crests between the main molar cusps are not well developed. While there are prominent shearing facets present on both the paracristid and protocristid notches, these surfaces are not flat and require wear to develop precise occlusal contact with the upper molars. There are additional wear facets present on the mesial face of paraconid and distal face of metaconid. As in other zhangheotheriids, interlocking between adjacent molars is not developed.

Upper dentition.—The alveoli and crowns for the full upper dentition are preserved on the left side. Crowns of the I1 and I2 are missing but their alveoli are large and circular in outline. Judging from the size of the alveoli, the first two are much larger than the I3 and I4. All the incisors are single rooted and the gaps between them decrease distally. The I3 has simple conical crown beveled anteriorly by wear, and tightly packed against the succeeding tooth. The crown of the I4 is damaged but appears to have been pyramidal in shape and larger than the I3.

A short diastema separates the upper canine from the I4. The crown of the canine is small and pyramidal in shape. There is a tiny, worn cuspule at the mesiolingual base of the tooth. The canine is likely double-rooted, but there remains some uncertainty as to how well-separated the roots are. There is a well-defined buccal groove running vertically along the visible part of root, suggesting that the roots split within the bone though the bases of the roots are fused. The buccal margin of the alveolus contours along this groove. The canine is lodged entirely within the maxilla.

A slightly longer diastema separates the canine from the P1. The P1 crown is missing on the left side but is well preserved on the right side (though the entire right tooth row has been shifted anteriorly by distortion of the specimen). The P1 is triangular and transversely compressed, and its crown is longer than and subequal in height to the canine. There is a low cuspule at the distal end of the crown and a slight basal bulge at the mesial end. The tooth is double-rooted. The P2 is preserved on both sides and is much smaller than the P1. Its main cusp is low, conical, and truncated by abrasion. There are two small cuspules present at the mesiobuccal base on the left side, though only one cuspule along a mesial cingulum is present on the right side. There is a slightly larger distobuccal cuspule present. The tooth appears to have a single heavy root, but there is a distinct groove on the buccal side suggesting that the root may split deeper in the bone (as appears to be likely for the upper canine). Due to its small size, it is possible that this tooth could be interpreted as a retained DP1, but based on the very limited evidence for replacement at this position in Mesozoic mammals we prefer a conservative approach.

The remaining three upper premolars are strongly molariform with well-developed cusp B and metacone becoming progressively more triangulated (with respect to the paracone)

distally. The crown of the P3 dwarfs the P2 and is twice the length of the P1. The paracone is tall and symmetrical, with the three principal cusps only slightly triangulated. A very weak buccal cingulum (=ectocingulum) connects small, paired cuspules on the mesial and distal ends of the crown; the larger of each pair is positioned slightly buccal to the mesiodistal axis of the tooth, and we consider these to be equivalent to the stylocone and metastyle of the molars. A weak lingual cingulum is only present distally, and is anchored to the cuspule positioned immediately distal to the metacone. The P4 is triangular in outline and the three main cusps form an angle of 165°. This tooth is the tallest in the postcanine series, even accounting for the incomplete eruption of the P5 (see below). Cusp B and the metacone are taller and better developed relative to those on the P3, and the stylocone is low and broad but larger than on the P3. There is a small cuspule present between the base of cusp B and the stylocone. This cuspule anchors the mesial portion of the lingual cingulum, which is better developed than that on the P3 but incomplete at the lingual base of the paracone. There is small cuspule present at the distal edge of the metacone but the metastylar corner of the crown is broken. The ectoflexus is very shallow.

The crown of the P5 is not fully erupted (the distal base of the crown has yet to clear the alveolar plane) and shows no wear on the cusps, supporting our identification of this tooth as a premolar instead of a molar. The main cusps on the crown are better triangulated with a much deeper ectoflexus than on the P3 and P4, with prominent buccally-projecting mesial and distal lobes, though the tooth is still longer than wide. Cusps B and the metacone are subequal and greater than half the height of the paracone, forming an angle of 135°. The stylocone and metastyle are well developed and conical. There is a tiny cuspule mesial and slightly lingual to the stylocone; while the position of this cuspule is consistent with a parastyle, no cusp is present

in this position on any of the molars and we consider it to be an anomalous feature of the premolars. The main distal crest, equivalent to the postmetacrista in therians, descends from the metacone and passes distal to the metastyle without joining the apex of this cusp. A weak notch is developed along the postmetacrista. The lingual cingulum is complete and slightly cuspidate, and is better developed than on the P4.

The M1 is distinguished from the P5 in that the main cusps are much better triangulated (95°) and the ectoflexus is much deeper. Cusp B and the metacone are both damaged or worn so their relative heights cannot be assessed. The stylocone and metastyle are prominent and conical but no parastyle or parastylar hook is developed. The main mesial shearing crest, equivalent to the preparacrista in therians but including cusp B in zhangheotheriids, passes mesial to the apex of the stylocone before curling distally to connect with this cusp. Two faint crests run from the apex of cusp B towards the ectoflexus, but fade before reaching the ectocingulum (these crests are present only on the left M1). The ectocingulum is weak and slightly crenulated. The lingual cingulum is broad except at the lingual-most point of the crown where it nearly pinches out; the cingulum bears several small cuspules, the largest of which is positioned between the paracone and cusp B. In occlusal view, the M1 is as long as wide. A well-developed shearing facet extends from the tip of cusp B to the stylocone. The facet does not extend onto the mesial face of the stylocone; instead, as the facet developed it excavated the lingual face of the stylocone and obliterated the mesial part of the lingual cingulum. Similar wear is developed from the metacone to the metastyle. However, unlike the mesial facet the distal wear facet extends all the way to the distobuccal edge of the crown. Additional wear facets are present on the lingual surfaces of cusp B and the metacone, which are continuous with small facets on the lingual cingulum. These facets match lower molar facets on the metaconid and paraconid, respectively. In general

morphology, the M2 and M3 are similar to the M1 except for the gradual distal reduction of the height of the metacone and the width of the metastylar lobe. The M3 is very asymmetrical in occlusal outline, though there is only a slight decrease in mesiodistal length of the molars distally.

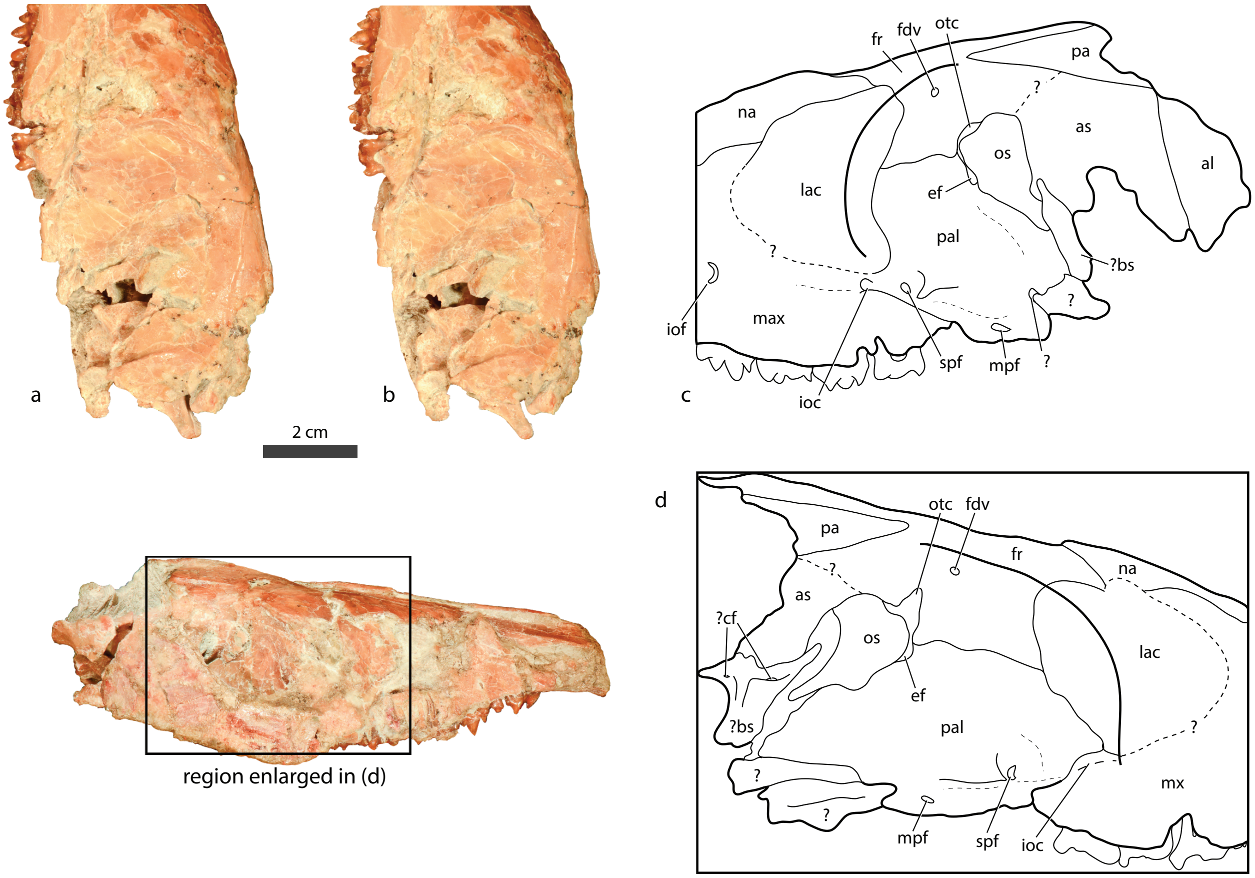

Supplemental Figure S1: Details of the orbital region and partial braincase of the zhangheotheriid *Anebodon luoi*, gen. et sp. nov., in left lateral (a–c) and right lateral (d) views. Illustrations are enlarged to show detail and are not to same scale as photographs. Abbreviations: al, anterior lamina of petrosal; as, alisphenoid; bs, basisphenoid; cf, carotid foramen; ef, ethmoidal foramen; fdv, foramen for frontal diploic vein; fr, frontal; ioc, infraorbital canal; iof, infraorbital foramen; lac, lacrimal; mpf, minor palatine foramen; mx, maxilla; na, nasal; os,

orbitosphenoid; otc, anterior opening of orbitotemporal canal; pa, parietal; pal, palatine; spf, sphenopalatine foramen.

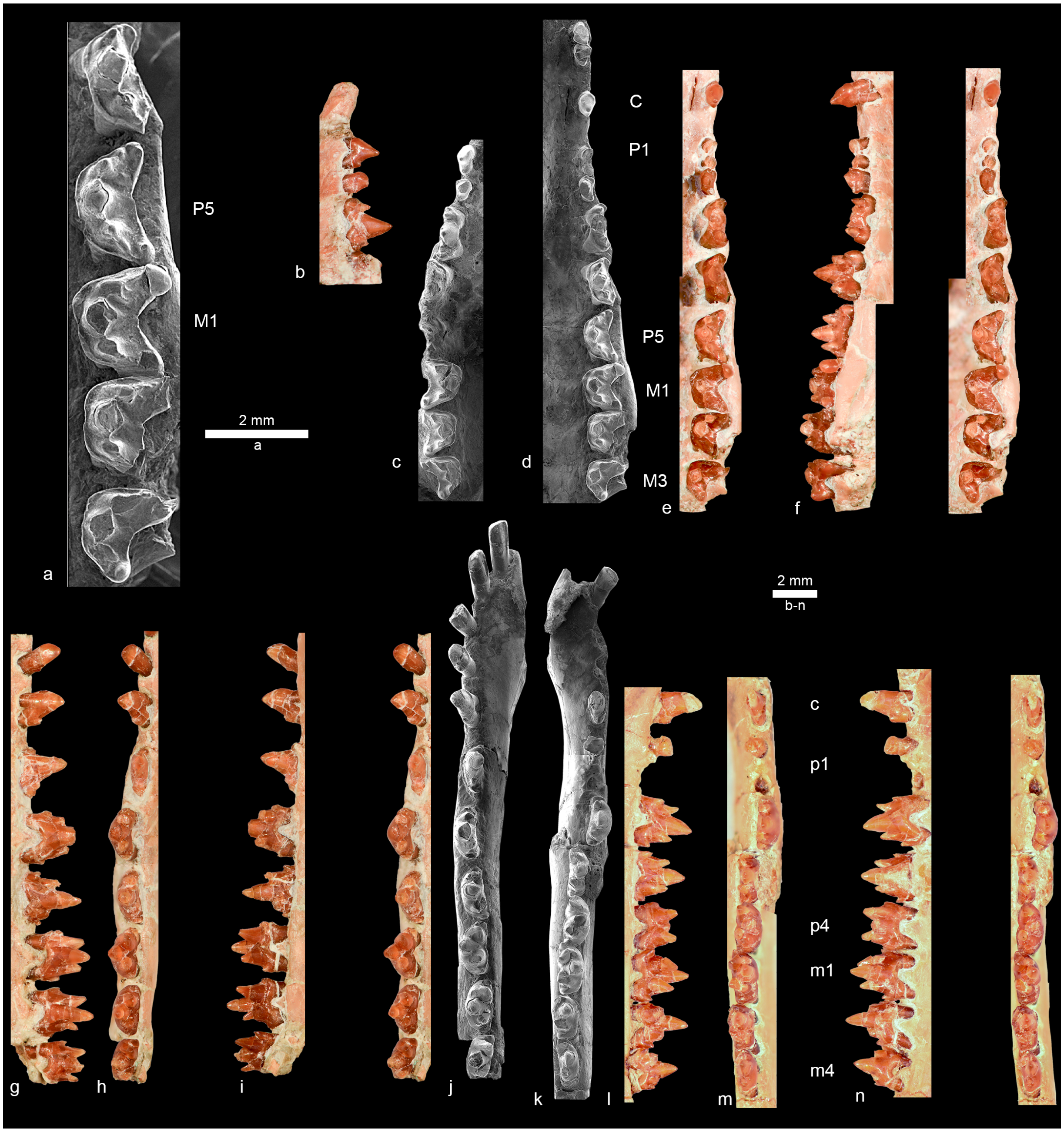

Supplemental Figure S2: Dentition of the zhangheotheriid *Anebodon luoi*, gen. et sp. nov. Right upper dentition in buccal (b, P1–P3 only) and occlusal (c, SEM) views; left upper dentition in occlusal (d,e: SEM and stereophotographs; a, enlargement of P4–M3) and buccal (f) views; left lower dentition (g–j) in buccal (g), occlusal (h,j: stereophotographs and SEM), and lingual (i)

views; right lower dentition (k–n) in occlusal (k,m: SEM and stereophotographs), lingual (l), and buccal (n) views.

List of characters (from Sweetman (2008)[1])

1. Prevallid (protoconid-paraconid) shearing surface at eruption:

(0) Interrupted: *Gobiotheriodon*, *Kuehneotherium*, *Maotherium*, *Tinodon*, *Zhangheotherium*;

(1) Continuous upon eruption: *Akidolestes*, *Heishanlestes*, *Spalacolestes*, *Spalacotheridium*, *Spalacotherium*, *Spalacotheroides*, *Symmetrodontoides*, *Symmetrolestes*, *Yaverlestes*;

(?) Unknown: *Morganucodon*.

Kiyatherium **0**

Anebodon **0**

2. Number of lower canine roots:

(0) One: *Akidolestes*, *Gobiotheriodon*, *Maotherium*, *Morganucodon*, *Kuehneotherium*, *Zhangheotherium*;

(1) Two: *Spalacotherium*, *Symmetrolestes*, *Tinodon*;

(?) Unknown: *Heishanlestes*, *Spalacolestes*, *Spalacotheridium*, *Spalacotheroides*, *Symmetrodontoides*, *Yaverlestes*.

Kiyatherium **0**

Anebodon **0**

3. Lower canine crown:

(0) Caniniform: *Akidolestes*, *Heishanlestes*, *Morganucodon*, *Spalacotherium*;

(1) Premolariform: *Tinodon*, *Zhangheotherium*;

(?) Unknown: *Gobiotheriodon*, *Kuehneotherium*, **Maotherium**, Spalacolestes, *Spalacotheridium*, *Spalacotheroides*, *Symmetrodontoides*, *Symmetrolestes*, *Yaverlestes*

***Kiyatherium* 1**

***Anebodon* 1**

4. Number of premolariforms:

(0) Four or more: *Akidolestes*, *Heishanlestes*, *Kuehneotherium*, *Morganucodon*, *Symmetrolestes*;

(1) Three or fewer: *Gobiotheriodon*, *Maotherium*, *Spalacotherium*, *Tinodon*, *Yaverlestes*, Zhangheotherium;

(?) Unknown: *Spalacolestes*, *Spalacotheridium*, *Spalacotheroides*, *Symmetrodontoides*.

***Kiyatherium* 0**

***Anebodon* 0**

5. Anterior molariform (m1) triangulation:

(0) No triangulation: *Morganucodon*;

(1) Obtuse triangulation: *Gobiotheriodon*, *Heishanlestes*, *Kuehneotherium*, *Maotherium*, *Spalacotherium*, *Tinodon*, *Zhangheotherium*;

(2) Acute triangulation: *Akidolestes*, *Spalacolestes*, *Spalacotheridium*, *Spalacotheroides*, *Symmetrodontoides*, *Symmetrolestes*, *Yaverlestes*.

***Kiyatherium* 1**

***Anebodon* 1**

6. Posterior molariform (m3 or more posterior) triangulation:

(0) No triangulation: *Morganucodon*;

(1) Obtuse triangulation: *Gobiotheriodon*, *Kuehneotherium*, *Tinodon*;

(2) Acute triangulation: *Akidolestes*, *Heishanlestes*, *Maotherium*, *Spalacolestes*, *Spalacotherium*, *Spalacotheridium*, *Spalacotheroides*, *Symmetrodontoides*, *Symmetrolestes*, *Yaverlestes*, *Zhangheotherium*.

***Kiyatherium* 2**

***Anebodon* 2**

7. Labial cingulid on lower molariform (scored for the penultimate lower molariform):

(0) Incomplete: *Akidolestes*, *Gobiotheriodon*, *Kuehneotherium*, *Maotherium*, *Morganucodon*, *Symmetrolestes*, *Tinodon*, *Zhangheotherium*;

(1) Complete or absent buccally only at the base of the protoconid: *Heishanlestes*, *Spalacolestes*, *Spalacotheroides*, *Spalacotheridium*, *Spalacotherium*, *Symmetrodontoides*, *Yaverlestes*.

***Kiyatherium* 0**

***Anebodon* 0**

8. Molariform interlock structure:

(0) Distal cuspule d fitting between cusps b and f of succeeding molariform: *Morganucodon*;

(1) Distal cuspule d fitting into the proximal cuspules e and f: *Kuehneotherium*, *Tinodon*;

(2) Distal cuspule d overlapping labially with the mesial cuspule e of the succeeding molar: *Akidolestes*, *Gobiotheriodon*, *Heishanlestes*, *Maotherium*, *Spalacolestes*, *Spalacotheridium*, *Spalacotherium*, *Spalacotheroides*, *Symmetrodontoides*, *Symmetrolestes*, *Yaverlestes*, *Zhangheotherium*.

Kiyatherium **2**

Anebodon **2**

9. Distal cingulid cuspule d of lower molariforms:

(0) Large: *Gobiotheriodon*, *Kuehneotherium*, *Morganucodon*;

(1) Small or absent: *Akidolestes*, *Heishanlestes*, *Maotherium*, *Spalacolestes*, *Spalacotheridium*, *Spalacotherium*, *Spalacotheroides*, *Symmetrodontoides*, *Symmetrolestes*, *Tinodon*, *Yaverlestes*, *Zhangheotherium*.

Kiyatherium **1**

Anebodon **1**

10. Paraconid and paracristid (scored for m2):

(0) Higher than the metaconid and protocristid: *Gobiotheriodon*, *Kuehneotherium*, *Maotherium*, *Spalacotherium*, *Symmetrolestes*, *Tinodon*, *Zhangheotherium*;

(1) Lower than the metaconid and protocristid: *Akidolestes*, *Heishanlestes*, *Spalacolestes*, *Spalacotheridium*, *Spalacotheroides*, *Symmetrodontoides*, *Yaverlestes*;

(?) Unknown: *Morganucodon*.

Kiyatherium **0**

Anebodon **0**

11. Paraconid (or cusp b) on posterior lower molariforms:

(0) In alignment with metaconid (or cusp c): *Akidolestes*, *Gobiotheriodon*,

Heishanlestes, *Kuehneotherium*, *Maotherium*, *Morganucodon*, *Spalacotheridium*, *Spalacotherium*, *Spalacotheroides*, *Symmetrolestes*, *Tinodon*, *Zhangheotherium*;

(1) Paraconid more labially positioned: *Spalacolestes*, *Symmetrodontoides*.

(?) Unknown: *Yaverlestes*.

***Kiyatherium* 0**

***Anebodon* 0**

12. Height of lower molariform crowns:

(0) Low crown: *Gobiotheriodon*, *Kuehneotherium*, *Maotherium*, *Morganucodon*, *Tinodon*, *Zhangheotherium*;

(1) High crown: *Akidolestes*, *Heishanlestes*, *Spalacolestes*, *Spalacotheridium*, *Spalacotherium*, *Spalacotheroides*, *Symmetrodontoides*, *Symmetrolestes*, *Yaverlestes*.

***Kiyatherium* 0**

***Anebodon* 0**

13. Preparacristid and postparacristid:

(0) Absent: *Gobiotheriodon*, *Kuehneotherium*, *Maotherium*, *Morganucodon*, *Spalacotherium*, *Tinodon*, *Zhangheotherium*;

(1) Present: *Akidolestes*, *Heishanlestes*, *Spalacolestes*, *Spalacotheridium*, *Spalacotheroides*, *Symmetrodontoides*, *Symmetrolestes*, *Yaverlestes*.

***Kiyatherium* 0**

***Anebodon* 0**

14. Upper molariforms:

(0) Retaining three distinctive cusps: *Akidolestes*, *Kuehneotherium*, *Maotherium*, *Morganucodon*, *Spalacotherium*, *Spalacotheroides*, *Tinodon*, *Yaverlestes*, *Zhangheotherium*;

(1) Compressed trigon without the three distinctive cusps: *Spalacolestes*, *Spalacotheridium*, *Symmetrodontoides*.

(?) Unknown: *Gobiotheriodon*, *Heishanlestes*, *Symmetrolestes*.

Kiyatherium **0**

Anebodon **0**

15. Distinctive B1 cusp on upper molariforms:

(0) Absent: *Kuehneotherium*, *Morganucodon*, *Spalacolestes*, *Spalacotheridium*, *Symmetrodontoides*, *Tinodon*, *Yaverlestes*;

(1) Present: *Maotherium*, *Spalacotherium*, *Spalacotheroides*, *Zhangheotherium*;

(?) Unknown: *Akidolestes*, *Heishanlestes*, *Gobiotheriodon*, *Symmetrolestes*.

Kiyatherium **1**

Anebodon **1**

16. Upper cusp C (‘metacone’):

(0) Present: *Akidolestes*, *Kuehneotherium*, *Maotherium*, *Morganucodon*, *Spalacotherium*, *Spalacotheroides*, *Tinodon*, *Yaverlestes*, *Zhangheotherium*;

(1) Absent: *Spalacolestes*, *Spalacotheridium*, *Symmetrodontoides*;

(?) Unknown: *Gobiotheriodon*, *Heishanlestes*, *Symmetrolestes*.

Kiyatherium **0**

***Anebodon* 0**

17. Stylocone:

(0) Large: *Kuehneotherium*, *Maotherium*, *Spalacotherium*, *Tinodon*, *Yaverlestes*, *Zhangheotherium*;

(1) Small: *Spalacolestes*, *Spalacotheridium*, *Symmetrodontoides*;

(?) Unknown: *Akidolestes*, *Gobiotheriodon*, *Heishanlestes*, *Morganucodon*, *Spalacotheroides*, *Symmetrolestes*.

***Kiyatherium* 0**

***Anebodon* 0**

18. Parastyle:

(0) Small: *Kuehneotherium*, *Maotherium*, *Spalacotherium*, *Tinodon*, *Zhangheotherium*;

(1) Large and hook-like: *Spalacolestes*, *Spalacotheridium*, *Spalacotheroides*, *Symmetrodontoides*, *Yaverlestes*.

(?) Unknown: *Akidolestes*, *Gobiotheriodon*, *Heishanlestes*, *Morganucodon*, *Symmetrolestes*.

***Kiyatherium* 0**

***Anebodon* ?**

19. Distal stylar cusp:

(0) Absent: *Kuehneotherium*, *Tinodon*;

(1) Present but not protruding distally: *Akidolestes*, *Maotherium*, *Spalacotherium*, *Spalacotheroides*, *Yaverlestes*, *Zhangheotherium*;

(2) Present and protruding: *Spalacolestes*, *Spalacotheridium*, *Symmetrodontoides*;

(?) Unknown: *Gobiotheriodon*, *Heishanlestes*, *Morganucodon*, *Symmetrolestes*.

***Kiyatherium* 1**

***Anebodon* 1**

20. Reduced and asymmetrical ultimate posterior upper molariforms:

(0) Absent: *Akidolestes*, *Maotherium*, *Spalacotherium*, *Spalacotheroides*, *Zhangheotherium*;

(1) Present: *Spalacolestes*, *Spalacotheridium*, *Symmetrodontoides*;

(?) Unknown: *Gobiotheriodon*, *Heishanlestes*, *Kuehneotherium*, *Morganucodon*, *Symmetrolestes*, *Tinodon*, *Yaverlestes*.

***Kiyatherium* 1**

***Anebodon* 0**

21. Internal cingulum on upper molariforms:

(0) Complete: *Kuehneotherium*, *Maotherium*, *Morganucodon*, *Spalacolestes*, *Spalacotheridium*, *Symmetrodontoides*;

(1) Incomplete: *Spalacotherium*, *Spalacotheroides*, *Tinodon*, *Yaverlestes*, *Zhangheotherium*;

(?) Unknown: *Akidolestes*, *Gobiotheriodon*, *Heishanlestes*, *Symmetrolestes*.

***Kiyatherium* 0**

***Anebodon* 0**

22. Extent of the pterygoid shelf:

(0) Absent: *Kuehneotherium*, *Morganucodon*;

(1) Present: *Akidolestes*, *Gobiotheriodon*, *Spalacotherium*, *Tinodon*, *Zhangheotherium*;

(2) Present and broad: *Heishanlestes*, *Spalacolestes*, *Spalacotheroides*, *Symmetrolestes*.

(?) Unknown: *Maotherium*, *Spalacotheridium*, *Spalacotherium*, *Symmetrodontoides*, *Yaverlestes*.

Kiyatherium **1**

Anebodon **?**

23. Deflected posteroventral border on the lateral side of the mandible:

(0) Absent: *Akidolestes*, *Gobiotheriodon*, *Kuehneotherium*, *Morganucodon*, *Tinodon*, *Zhangheotherium*;

(1) Present: *Heishanlestes*, *Spalacolestes*, *Symmetrolestes*, *Yaverlestes*.

(?) Unknown: *Maotherium*, *Spalacotheridium*, *Spalacotherium*, *Spalacotheroides*, *Symmetrodontoides*.

Kiyatherium **0**

Anebodon **?**

24. Meckel's groove:

(0) Present: *Gobiotheriodon*, *Kuehneotherium*, *Morganucodon*, ***Maotherium***, *Spalacotherium*, *Symmetrolestes*, *Tinodon*, *Zhangheotherium*;

(1) Absent: *Heishanlestes*, *Spalacolestes*, *Symmetrodontoides*, *Yaverlestes*;

(?) Unknown: *Akidolestes*, *Spalacotheridium*, *Spalacotheroides*.

Kiyatherium **0**

Anebodon **0**

25. Coronoid facet:

(0) Present: *Heishanlestes*, *Kuehneotherium*, *Morganucodon*, *Zhangheotherium*;

(1) Absent: *Spalacolestes*, *Spalacotherium*, *Symmetrolestes*;

(?) Unknown: *Akidolestes*, *Gobiotheriodon*, *Maotherium*, *Spalacotheridium*, *Spalacotheroides*, *Symmetrodontoides*, *Tinodon*, *Yaverlestes*.

Kiyatherium **?**

Anebodon **?**

26. Size of coronoid process:

(0) Broad: *Heishanlestes*, *Kuehneotherium*, *Morganucodon*, *Spalacolestes*, *Spalacotherium*, *Tinodon*;

(1) Narrow and gracile: *Akidolestes*, *Maotherium*, *Symmetrolestes*, *Zhangheotherium*;

(?) Unknown: *Gobiotheriodon*, *Spalacotheridium*, *Spalacotheroides*, *Symmetrodontoides*, *Yaverlestes*.

Kiyatherium **1**

Anebodon **1**

27. Position of the dentary condyle:

(0) Below or level with the alveolar margin: *Kuehneotherium*, *Morganucodon*;

(1) Above the alveolar level: *Akidolestes*, *Heishanlestes*, *Maotherium*, *Spalacotherium*, *Symmetrolestes*, *Zhangheotherium*;

(?) Unknown: *Gobiotheriodon*, *Spalacolestes*, *Spalacotheridium*, *Spalacotheroides*, *Symmetrodontoides*, *Tinodon*, *Yaverlestes*.

***Kiyatherium* 1**

***Anebodon* ?**

28. Postdentary trough:

(0) Present: *Kuehneotherium*, *Morganucodon*;

(1) Absent: *Akidolestes*, *Gobiotheriodon*, ***Maotherium***, *Heishanlestes*, *Spalacotherium*, *Spalacotheroides*, *Symmetrolestes*, *Tinodon*, *Zhangheotherium*;

(?) Unknown: *Spalacolestes*, *Spalacotheridium*, *Symmetrodontoides*, *Yaverlestes*.

***Kiyatherium* 1**

***Anebodon* ?**

29. Number of lower molariforms:

(0) Five or fewer: *Gobiotheriodon*, *Morganucodon*, *Symmetrolestes*, *Tinodon*, *Yaverlestes*;

(1) Six or more: *Akidolestes*, *Heishanlestes*, *Kuehneotherium*, *Maotherium*, *Spalacolestes*, *Spalacotheridium*, *Spalacotherium*, *Symmetrodontoides*, *Zhangheotherium*;

(?) Unknown: *Spalacotheroides*.

***Kiyatherium* 0**

***Anebodon* 0**

30. Ultimate premolar crown:

(0) premolariform; *Morganucodon*, *Akidolestes*, *Heishanlestes*, *Spalacolestes*, *Spalacotheridium*, *Spalacotherium*, *Spalacotheroides*, *Symmetrodontoides*,

(1) molariform: *Tinodon*, *Maotherium*, *Zhangheotherium*, *Symmetrolestes*;

(?) Unknown: *Yaverlestes*, *Gobiotheriodon*, *Kuehneotherium*.

***Kiyatherium* 1**

***Anebodon* 1**

References

1 Sweetman, S. C. A spalacolestine spalacotheriid (Mammalia, Trechnotheria) from the Early Cretaceous (Barremian) of southern England and its bearing on spalacotheriid evolution. *Palaeontology* **51**, 1367-1385 (2008).

2 Rougier, G. W. *Vincelestes neuquenianus Bonaparte (Mammalia, Theria) un Primitivo Mamífero del Cretácico Inferior de la Cuenca Neuquina*, Universidad PhD Dissertation Nacional de Buenos Aires, (1993).

3 Lillegraven, J. A. & Krusat, G. Cranio-mandibular anatomy of *Haldanodon exspectatus* (Docodonta; Mammalia) from the Late Jurassic of Portugal and its implications to the evolution of mammalian characters. *Contrib. Geol. Univ. Wyo.* **28**, 39-138 (1991).

4 Rougier, G. W., Ji, Q. & Novacek, M. J. A new symmetrodont mammal with fur impressions from the Mesozoic of China. *Acta Geol. Sin.(Engl.)***77**, 7-14 (2003).

5 Ji, Q., Luo, Z.-X., Zhang, X., Yuan, C.-X. & Xu, L. Evolutionary development of the middle ear in Mesozoic therian mammals. *Science* **326**, 278-281 (2009).

6 Wible, J. R. & Rougier, G. W. The cranial anatomy of *Kryptobaatar dashzevegi* (Mammalia, Multituberculata), and its bearing on the evolution of mammalian characters. *Bull. Am. Mus. Nat. Hist.* **247**, 1-124 (2000).

7 Kermack, K. A., Mussett, F. & Rigney, H. W. The skull of *Morganucodon*. *Zool. J. Linn. Soc.* **71**, 1-158 (1981).

8 MacPhee, R. D. E. Morphology, adaptations, and relationships of *Plesiorycteropus* : and a diagnosis of a new order of eutherian mammals. *Bull. Am. Mus. Nat. Hist.* **220**, 1-214 (1994).

9 Sánchez-Villagra, M. R. & Wible, J. R. Patterns of evolutionary transformation in the petrosal bone and some basicranial features in marsupial mammals, with special reference to didelphids. *J. Zool. Syst. Evol. Res.* **40**, 26-45, doi:10.1046/j.1439-0469.2002.00173.x (2002).

10 Hopson, J. A. & Rougier, G. W. Braincase structure in the oldest known skull of a therian mammal: implications for mammalian systematics and cranial evolution. *Am. J. Sci.* **293**, 268-299 (1993).
